# Development and validity of a booklet on myocardial revascularization for patient and caregiver education

**DOI:** 10.1590/0034-7167-2024-0446

**Published:** 2025-12-12

**Authors:** Vanêssa Piccinin Paz, Maria de Fátima Mantovani, Elisiane Lorenzini, Ângela Tais Mattei da Silva, Robson Giovani Paes, Catiele Raquel Schmidt

**Affiliations:** I Universidade Federal do Paraná. Curitiba, Paraná, Brazil; II Universidade Federal de Santa Catarina. Florianópolis, Santa Catarina, Brazil; III Conselho Regional de Enfermagem. Londrina, Paraná, Brazil

**Keywords:** Adult Health, Cardiac Surgical Procedures, Validation Study, Health Education, Myocardial Revascularization., Salud del Adulto, Procedimientos Quirúrgicos Cardíacos, Estudio de Validación, Educación en Salud, Revascularización Miocárdica.

## Abstract

**Objectives:**

to develop and validate a booklet with care guidelines for patients undergoing cardiac surgery.

**Methods:**

methodological research developed in four stages: situational assessment -interviews with 24 patients undergoing cardiac surgery in the last half of 2019; theoretical procedure - literature analysis; experimental procedure - booklet development; and analytical procedure - content and appearance validity using the Delphi technique. Descriptive statistics, Content Validity Index measurement, and Cronbach’s alpha Wilcoxon test were used for analysis.

**Results:**

the booklet provides guidance for all phases of surgery and home care. The Content Validity Index was 94.6%. Modifications were made to the images and text were adapted, based on judges’ suggestions.

**Conclusions:**

the booklet provides guidance for people undergoing cardiac surgery and their caregivers. Its content and appearance demonstrate evidence of validity for use by this target audience.

## RESUMEN

**Objetivos:**

desarrollar y validar un folleto con pautas de atención para pacientes sometidos a cirugía cardíaca.

**Métodos:**

investigación metodológica desarrollada en cuatro etapas: evaluación situacional - entrevistas a 24 pacientes sometidos a cirugía cardíaca en el segundo semestre de 2019; procedimiento teórico - análisis de la literatura; procedimiento experimental - desarrollo del folleto; y procedimiento analítico - validación de contenido y apariencia mediante la técnica Delphi. Se utilizaron estadísticas descriptivas, el Índice de Validez de Contenido y la prueba alfa de Cronbach-Wilcoxon para el análisis.

**Resultados:**

el folleto ofrece orientación para todas las fases de la cirugía y la atención domiciliaria. El Índice de Validez de Contenido fue del 94,6 %. Se modificaron las imágenes y se adaptó el texto según las sugerencias del jurado.

**Conclusiones:**

el folleto ofrece orientación a las personas que se someten a cirugía cardíaca y a sus cuidadores. Su contenido y presentación demuestran su validez para este público objetivo.

## INTRODUCTION

Cardiovascular diseases are one of the main causes of morbidity and mortality in Brazil and globally^(1)^, with coronary artery disease (CAD) being considered the main cause of deaths in the country between 1990 and 2019. CAD treatment involves procedures such as coronary angioplasty and surgical procedures^(2)^.

From 2008 to 2019, there was a 64% increase in surgical procedures related to cardiovascular diseases in the public health system and a 54% increase in hospitalizations for acute myocardial infarction (AMI). A reduction in these conditions was observed in older adults and an increase in adults aged 15 to 49 years^(3)^.

Coronary artery bypass graft (CABG) surgery is usually the result of a sudden event. Therefore, when patients undergoing CABG return home after discharge, they often experience physical and emotional changes associated with insecurity about the possibility of a recurrence of heart attack and complications inherent to the surgery. The discharge process should be planned during hospitalization, with systematic guidance from the nursing team^(4)^. However, the ability to interpret their health condition and apply the guidance received can influence self-care, thus reducing possible problems^(5)^.

It is noteworthy that educational materials have proven effective in promoting care for other chronic diseases, such as hypertension and diabetes mellitus^(6,7)^. Such resources encourage active participation in treatment, as found in a quasi-experimental study that used a health literacy (HL)-based intervention with people about diabetes^(8)^.

Reports from people after a heart attack indicate a lack of clarity in self-care at home, highlighting deficiencies in the health education offered by services^(9,10)^, a fact that highlights the relevance of guidance and monitoring for these patients.

A Pakistani study of 50 post-AMI patients found that 80% of the sample was literate and 46% had satisfactory knowledge about AMI. After two weeks of guidance, the results reinforce the effectiveness (p<0.001) in practice, attitudes, and understanding, demonstrating the positive effect of the guidance^(11)^.

Knowledge about the health-disease process, motivation, self-efficacy, social support, and understanding of clinical condition are considered key elements for improving self-management skills^(12)^, especially after surgery. A person’s involvement in care, through understanding their illness, willingness to change lifestyle, and having a support network, directly impacts the effectiveness of healthcare^(13)^.

Thus, the use of educational technologies represents an effective strategy for expanding access to information about health conditions and the care required, fostering people’s understanding and proactive role in the recovery process. In the context of cardiac surgery, although educational materials focused on preand postoperative care exist in the literature, no booklets covering all stages of the surgical procedure, including guidance for both patients and their caregivers, were identified. Therefore, this justifies the development of this research, which began with the following questions: what elements should be included in an educational booklet with guidelines for the care of patients undergoing cardiac surgery? Does the booklet present evidence of content and appearance validity?

## Study relevance

This article is derived from the doctoral thesis entitled “Nurses and Health Literacy in the Self-Management of Care for People After Cardiac Surgery”^(14)^, defended at the Federal University of Paraná. It highlights the importance of educational technologies grounded in health literacy for supporting the care of patients undergoing myocardial revascularization and their caregivers.

## OBJECTIVES

To create and validate a booklet with care guidelines for people undergoing cardiac surgery.

## METHODS

This methodological study was conducted in four distinct phases: identification of needs through situational assessment; theoretical foundation; development and experimental validity of educational materials; and analysis of results^(15)^.

The study was conducted between December 2020 and June 2021 in the western region of the state of Paraná, Brazil, in accordance with the Revised Standards for Quality Improvement Reporting Excellence^(16)^ guidelines, linked to the EQUATOR network.

This research comprises a doctoral thesis entitled “*O enfermeiro e a literacia em saúde na autogestão do cuidado de pessoas pós-cirurgias cardíacas*”^(14)^. The research was approved by the Research Ethics Committee (Opinion 5,048,079), and all participants, including expert evaluators, signed the Informed Consent Form.

### Sample, inclusion and exclusion criteria

Initial data collection was performed through document analysis of clinical records archived in the Medical and Statistical Archives Service (In Portuguese, *Serviço de Arquivo Médico e Estatístico* - SAME) of a referral cardiology hospital in the municipality where the study was conducted. The records of patients diagnosed with AMI and undergoing CABG between July and December 2019 were included, according to pre-established criteria. All cases that met the defined criteria comprised the final sample, consisting of 24 participants.

In the subsequent stage, Brazilian and international publications in Portuguese, English, and Spanish were included, with no time limit and covering all stages of CABG. Editorials, books, opinion pieces, dissertations, theses, and duplicate publications were excluded. No articles meeting the inclusion criteria were identified.

The next stage was the booklet creation, which was based on the testimonies of people undergoing CABG surgery in the last six months of 2019. After transcribing the testimonies, the main difficulties mentioned were grouped together, serving as a basis for developing guidelines based on scientific literature.

In the final stage, expert judges (masters and doctors) and technical judges (multidisciplinary team of the cardiology hospital) were selected, totaling at least six judges^(17)^. Experts were selected through snowball sampling, and inclusion criteria were having at least four years of professional experience (clinical, teaching, and/or research), a doctoral or master’s degree in nursing, and being a resident of the country. These criteria were assessed based on data from the Lattes CV. Technicians working in the institution’s coronary care unit were included. Judges who did not respond to the electronic form within the specified deadline were excluded.

### Study protocol

The study was developed in four stages: (1) situational diagnosis; (2) theoretical procedure; (3) experimental procedure; and (4) analytical procedure^(14)^.

The first stage identified the needs and/or challenges of individuals undergoing CABG. This stage was conducted in the first half of December 2020 at a referral cardiology hospital in western Paraná, within the SAME.

After applying eligibility criteria, data collection was conducted at home using an instrument that addressed questions about individuals’ perceptions of CABG, challenges faced preand post-surgery, instructions/guidance received, discharge preparation, and the home health team’s care segment.

The interviews were recorded, transcribed, and subjected to thematic coding analysis using the *Interface de R pour les Analyses Multidimensionnelles de Textes et de Questionnaires* 0.6-alpha 3 software. This process aimed to identify the main needs identified by participants and inform the booklet development. This phase followed the of the COnsolidated criteria for Reporting Qualitative Research guidelines^(18)^.

The second stage, an integrative literature review, took place in July 2021, and for its implementation, the Preferred Reporting Systematic Reviews and Meta-Analyses of Studies^(19)^ recommendations were followed in the Medical Literature Analysis and Retrieval System Online, Scientific Electronic Library Online, *Banco de Dados em Enfermagem* and *Literatura Latino-Americana e do Caribe em Ciências da Saúde* databases. After selection based on eligibility criteria, publications were assessed by reading the title and abstract to verify whether they met the objective of the review.

In the third stage, the booklet was developed, conceived as an educational support technology, with the purpose of facilitating learning and reinforcing the verbal information provided by the health team^(20)^. The material textual construction was guided by the needs and difficulties reported by participants in interviews, which subsidized the definition of contents based on scientific literature.

The fourth stage consisted of content validity assessment using a modified online Delphi technique, with a consensus meeting and a maximum of three rounds of assessments. In this stage, the form proposed by Rodrigues^(21)^ was used, adapted to the research theme, which included aspects of objective, structure, presentation, and relevance. For technical experts, the form included questions about content assessment, language, graphic illustration, motivation, and cultural appropriateness. In each domain assessed, judges could make considerations if they deemed necessary.

Responses followed a Likert-type scale format. For expert judges, the Likert scale consisted of five response options: 1 - strongly disagree; 2 - partially agree; 3 - neither agree nor disagree; 4 - agree; and 5 - strongly agree. For technical judges, the Likert scale had three options: 0 - inadequate; 1 - partially adequate; and 2 - adequate. The developed instruments were transposed into online forms, which enabled simultaneous access and assessment.

The data were transcribed into a Microsoft Excel^®^ document and subsequently analyzed using Content Validity Index (CVI), mean, standard deviation, and Cronbach’s alpha. To assess expert judges’ CVI, responses 1, 2, and 3 were grouped as “do not agree”, and responses 4 and 5 as “agree”. Technical judges’ responses were assessed by considering the total sum of points and dividing these by the total number of items in the questionnaire. The material was considered adequate when 100% of experts agreed with the item^(22)^. To meet the institution’s/hospital routine’s needs, we chose to use two scales with different scores to assess the instrument’s validity.

**Figure 1:**
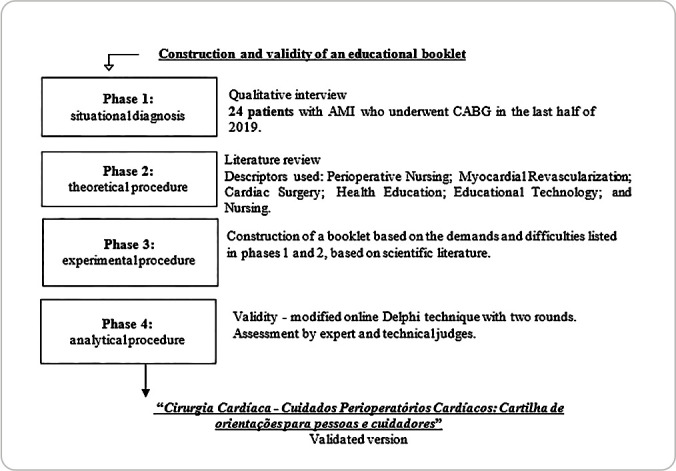
Educational booklet development and validity phases, Curitiba, Paraná, Brazil, 2023

Cronbach’s alpha values ≥ 0.60 represent moderate internal consistency^(23)^. The booklet was considered validated when each item obtained a CVI^(23)^ equal to or greater than 0.8. The Wilcoxon test was used to compare the first and second rounds. Figure 1 shows the research phases^(16)^.

## RESULTS

Twenty-four people participated in the first stage, 18 (75%) men aged between 44 and 68 years, and 14 (58.3%) with less than nine years of education. The transcription of interviewees’ audio recordings had a 90.23% success rate, generating 24 texts. Content categorization revealed questions related to postoperative eating habits, as well as information gaps in the transition to home care, especially regarding self-care and rehabilitation guidelines.

The literature search yielded 43 articles, 40 of which were ineligible. Three articles were read in full and subsequently excluded: two for addressing nursing care in the postoperative period after cardiac surgery in the Intensive Care Unit and anxiety; and the third for identifying information that hospitalized patients undergoing CABG believed was relevant to receive before hospital discharge regarding self-care^(24)^.

Thus, an integrative review demonstrated the need for educational materials that covered all phases of the surgical process: from preoperative care to home rehabilitation. This resulted in the development of the proposed educational materials, based fundamentally on the needs identified by those who experienced the surgical process and the challenges faced by them and their caregivers during rehabilitation.

The booklet was created using guidelines proposed by the Ministry of Health and the Brazilian Society of Cardiology, including text development, illustrations, and layout. A journalist and a graphic designer participated in this process. The material was produced on standard A4 paper size, making it easier to handle and read. The font used was Lato, size 10, regular weight, line height 8 and paragraph height 16, and justified alignment. The primary color of the booklet was red, with variations to light pink; the secondary color was green, with variations to light green; the quaternary color was brown, with variations to light brown; and the neutral colors were black, white, and gray. After layout, the booklet was sent for review by experts, judges, and technicians.

The editing was guided by the target audience’s interests. The goal was to create straightforward texts using clear and accessible language. The version of the booklet submitted by the authors for validity was titled “*Cirurgia Cardíaca - Cuidados Perioperatório Cardíacos: Cartilha de orientações para pessoas e cuidadores*”.

Content validity was assessed by seven experts, the majority of whom were female (85.7%), had doctorates (57.1%), and master’s degrees in nursing (42.9%). The technical panel consisted of a nursing technician working in the department where the research was conducted and a physical therapist specializing in intensive cardiac rehabilitation. Nurses and physicians did not respond.

After analyzing the material, technical experts found it 100% adequate in all aspects. Therefore, a second round of assessment was not conducted with participants. Table 1 presents the results related to agreement among expert judges.

**Table 1:** Material content validity process. Curitiba, PR, Brazil, 2023

Assessed items	Questions	1^st^ round			2^nd^ round		
		Mean	Standard deviation	CVI (%)	Mean	Standard deviation	CVI (%)
Content	1 - Is the information/content consistent with the daily care needs of people after cardiac surgery?	3.9	1.3	71.4	4.7	0.5	100
	2 - The information (and content) is important for the quality of care for people after cardiac surgery.	4.0	1.7	71.4	4.6	0.5	100
	3 - The information from educational technology encourages and/or encourages changes in patient behavior and attitudes toward post-cardiac surgery care.	4.3	1.1	85.7	4.4	0.5	100
	4 - It can be circulated within scientific circles in the field.	4.7	0.5	100	4	1.0	86
	5 - It meets the objectives of institutions that care for people undergoing cardiac surgery.	4.4	0.5	100	4.7	0.4	100
Structure and presentation	6 - The booklet is appropriate for people undergoing cardiac surgery.	4.1	1.1	85.7	4.6	0.5	100
	7 - The messages are presented clearly and objectively.	4.0	1.4	71.4	4.3	1.1	86
	8 - The information presented is scientifically accurate.	4.7	0.5	100	4.4	0.5	100
	9 - The material is appropriate for the sociocultural level of care for people after cardiac surgery.	3.3	1.4	42.9	4.7	0.5	100
	10 - There is a logical sequence of proposed content.	4.7	0.5	100	4.7	0.5	100
	11 - The information is well-structured in terms of grammar and spelling.	3.0	1.3	42.9	3.4	1.5	57
	12 - The writing style corresponds to the level of knowledge of patients and their families.	3.0	1.3	42.9	4.3	0.5	100
	13 - The information on the cover, back cover, summary, acknowledgments, and/or introduction is coherent.	3.1	1.5	42.9	4.4	0.5	100
	14 - The title and topic sizes are appropriate.	4.4	0.5	100	4.1	1.1	100
	15 - The illustrations are expressive and sufficient.	4.0	1.0	85.7	4	1	86
	16 - The number of pages is appropriate.	4.1	1.1	85.7	4.3	0.5	100
Relevance	17 - The topics portray key aspects that should be reinforced.	4.4	0.5	100	4.6	0.5	100
	18 - The material allows for the transfer and generalization of learning to different contexts (hospital and home).	4.7	0.5	100	4.4	0.5	100
	19 - The booklet promotes knowledge building.	4.7	0.5	100	4.6	0.5	100
	20 - The material addresses the topics necessary for people to understand after cardiac surgery.	4.7	0.5	100	4.4	0.5	100
	21 - The booklet is suitable for use by anyone who has undergone cardiac surgery.	4.4	0.5	100	4	1	86
General CVI				82.3			94.6

*Legend: Wilcoxon test (p-value) = 0.052; CVI - Content Validity Index.*

Cronbach’s alpha in the second round was 0.93, representing a significant result compared to expert judges. Regarding CVI, the overall results were 83.3% and 94.6% in the first and second rounds, respectively. The judges’ main suggestions are shown in Chart 1 and were related to questions 9, 11, 12, and 13.

**Chart 1:** Content expert recommendations provided in the first round of validity, Curitiba, Paranpa, Brazil, 2023

Question	Item analyzed	Recommendation
9	The material is appropriate for the sociocultural level of care for people after cardiac surgery.	Review technical terminology.
11	The information is well-structured in terms of grammar and spelling.	Review spelling and grammar.
12	The writing style corresponds to the knowledge level of the patients and their families.	Review technical terms.
13	The information on the cover, back cover, summary, acknowledgments, and/or introduction is consistent.	Review colors used and make changes to the title and index.

After adjustments, a new analysis was carried out by seven judges, and only question 11 required revision in terms of spelling and grammatical agreement, without interfering with the result of the general CVI, which indicated good content validity with a value of 94.6%.

## DISCUSSION

The booklet aims to minimize knowledge gaps and inform people undergoing CABG and during recovery, as well as providing support for the healthcare team. The aspects considered by people undergoing late CABG were similar to those reported in other studies^(24,25)^.

The booklet development was guided by users’ own perceptions, giving the material a personalized approach aligned with the real challenges faced post-CABG surgery. Thus, based on the accounts of people undergoing late CABG and their caregivers, educational material was developed that met the needs of this population, based on the literature.

It should be noted that, in late rehabilitation, issues related to the identification of risk factors, psychosocial risk, and support were highlighted. Ineffective communication with healthcare services emerged as a factor in the distancing of the relationship between patients and the health team.

During late rehabilitation, issues related to emotional lability and limitations imposed by CABG, as well as fear of a new AMI, stood out, even a year after the event. Having an individualized intervention plan allows for the definition of strategies that facilitate care delivery, especially when combined with health education. This combination promotes treatment adherence, expands patient knowledge, improves self-care, and contributes to understanding the procedure through clear and well-founded information^(26,27)^.

According to American research, educational materials focused on cardiovascular health present textual complexity that is greater than the target audience’s ability to understand, negatively impacting the interpretation of information and hindering its application in decision-making related to self-care^(28)^.

Obstacles related to accessing secure data and interpreting communicated information hinder effective HL. Therefore, it is crucial to create teaching materials in understandable language and with relevance to the target audience to minimize these obstacles and make the content understandable^(28)^.

The level of HL influences the understanding of information, the ability to perform care-related actions, commitment to the therapeutic plan, and the acceptance of health promotion actions^(29,30)^. It represents a key factor for changing lifestyle and promoting more favorable health outcomes, in addition to being a determining factor for modifying habits and, consequently, for achieving better health-related outcomes^(31,32)^.

Therefore, the adequacy and clarity of information, combined with the healthcare team’s communication skills, are essential for people with low levels of HL, and the use of healthcare technologies can aid this process^(33)^. In this sense, a scoping review sought to classify and map the characteristics and interventions that make the healthcare team responsive to patients’ HL, identifying 19 strategies, highlighting the use of educational materials^(34)^. Therefore, the creation and dissemination of educational materials aimed at post-CABG patients and their caregivers is a teaching resource that contributes to increasing the level of HL by presenting guidance objectively, using accessible language, and using images.

The Delphi technique used for content validity allowed remote expert participation^(35)^, resulting in the development of effective, practical, and reliable educational materials.

The booklet can stimulate learning, contribute to post-surgery recovery, and support the challenges of rehabilitation. There was consensus that the material is suitable for healthcare education, as it presents accessible, relevant content, and an appropriate format^(36)^.

The expert assessment process provides methodological rigor to the construction of educational content, contributing to its improvement and ensuring greater effectiveness in relation to its intended purpose^(37,38)^.

### Study limitations

The limiting factor in this research was obtaining data restricted to a local healthcare service, referring to the needs of those people undergoing CABG.

### Contributions to nursing, health, or public policy

The material resulting from this research addresses the main topics that should be emphasized during guidance for people who will be and/or have undergone myocardial revascularization and their caregivers, which were listed based on patients’ needs and validated by professionals specialized in the subject.

The booklet can be incorporated into nurses’ clinical practice during nursing consultations, serving as a support tool for quick and qualified guidance and contributing to the reduction of complications in the postoperative period of cardiac surgery. The development and validity of educational materials in healthcare settings are essential, as they provide reliable information that helps patients resolve their doubts.

## CONCLUSIONS

Qualified listening to patients undergoing CABG, combined with theoretical foundations based on literature, resulted in the development of a booklet whose content was validated by experts. The educational technology developed demonstrated evidence of content validity and is recommended for use by healthcare professionals, with an emphasis on nursing practice. Its use also proves appropriate in the context of care provided between family members and patients, as it includes guidance from the preoperative period through the home rehabilitation phase.

The validity of this booklet by specialists and the healthcare team during its development was found to qualify it for use in the institution. The development of the material demonstrates promising results related to the care provided to individuals undergoing CABG. Depending on the rigor of the validity process and the necessary adjustments, the booklet has the potential to be implemented in different care settings that serve individuals with this clinical profile.

## Data Availability

The research data are available in a repository: https://doi.org/10.5281/zenodo.17214413.
