# Supplementary material for: Development and validity of a booklet on myocardial revascularization for patient and caregiver education
Source: Rev Bras Enferm. 2025 Dec 12;78(Suppl 4):e20240446. doi: 10.1590/0034-7167-2024-0446 (PMC12707763; doi:10.1590/0034-7167-2024-0446)
Supplement: Translation

# Translation: Construção e validação de cartilha sobre revascularização miocárdica para educação de pacientes e cuidadores

**DOI:** 10.1590/0034-7167-2024-0446pt

**Authors:** Vanêssa Piccinin Paz, Maria de Fátima Mantovani, Elisiane Lorenzini, Ângela Tais Mattei da Silva, Robson Giovani Paes, Catiele Raquel Schmidt

**Affiliations:** I Universidade Federal do Paraná. Curitiba, Paraná, Brasil; II Universidade Federal de Santa Catarina. Florianópolis, Santa Catarina, Brasil; III Conselho Regional de Enfermagem. Londrina, Paraná, Brasil

**Keywords:** Saúde do Adulto, Procedimentos Cirúrgicos Cardíacos, Estudo de Validação, Educação em Saúde, Revascularização Miocárdica.

## Abstract

**Objetivos:**

construir e validar uma cartilha com orientações de cuidados para pessoas submetidas à cirurgia cardíaca.

**Métodos:**

pesquisa metodológica desenvolvida em quatro etapas: diagnóstico situacional - entrevistas com 24 pessoas submetidas a cirurgia cardíaca no último semestre de 2019; procedimento teórico - análise da literatura; procedimento experimental - elaboração da cartilha; e procedimento analítico - validação de conteúdo e aparência pela técnica Delphi. Para análise, utilizaram-se estatística descritiva, mensuração do Índice de Validade de Conteúdo e teste de Wilcoxon alfa de Cronbach.

**Resultados:**

a cartilha contempla orientações em todas as fases da cirurgia e cuidados domiciliares. O Índice de Validade de Conteúdo foi de 94,6%. Foram realizadas modificações nas imagens e adequação de textos, conforme sugestões dos juízes.

**Conclusões:**

a cartilha abrange orientações para pessoas submetidas à cirurgia cardíaca e seus cuidadores. Seu conteúdo e aparência têm evidências de validade para utilização por este público-alvo.

## INTRODUÇÃO

As enfermidades cardiovasculares figuram como uma das principais causas de morbimortalidade brasileira e globalmente^(1)^, com a doença arterial coronariana (DAC) sendo considerada a principal responsável por mortes no país entre 1990 e 2019. O tratamento da DAC envolve procedimentos como angioplastia coronariana e procedimentos cirúrgicos^(2)^.

De 2008 a 2019, observou-se um crescimento de 64% nos procedimentos cirúrgicos relacionados a doenças cardiovasculares no sistema público de saúde e de 54% nas internações por infarto agudo do miocárdio (IAM). Observaram-se redução dessas condições em idosos e aumento em adultos de idade de 15 a 49 anos^(3)^.

A cirurgia de revascularização do miocárdio (CRVM) geralmente é decorrente de um evento súbito. Desta forma, quando as pessoas submetidas à CRVM retornam à residência após a desospitalização, é comum que apresentem alterações físicas e emocionais associadas à insegurança diante da possibilidade de recorrência do infarto e de complicações inerentes à cirurgia. O processo de alta deve ser planejado durante a internação, com orientações sistematizadas da equipe de enfermagem^(4)^. Contudo, a capacidade de interpretar sua condição de saúde e aplicar as orientações recebidas pode influenciar o autocuidado, reduzindo, portanto, os possíveis agravos^(5)^.

Salienta-se que os materiais educativos têm se mostrado eficazes na promoção de cuidados voltados a outras doenças crônicas, como hipertensão arterial sistêmica e diabetes *mellitus* ^(6,7)^. Tais recursos favorecem a participação ativa no tratamento, conforme constatado em pesquisa de delineamento quase-experimental que utilizou intervenção baseada no letramento em saúde (LS) com pessoas acerca do diabetes^(8)^.

Relatos de pessoas pós-infarto indicam falta de clareza no autocuidado em domicílio, apontando deficiências na educação em saúde ofertada pelos serviços^(9,10)^, fato que evidencia a relevância das orientações e acompanhamento desses pacientes.

Estudo paquistanês realizado com 50 pessoas pós-IAM identificou que 80% da amostra era alfabetizada e 46% possuíam conhecimentos satisfatório sobre o IAM. Após orientações realizadas por duas semanas, os resultados reforçam a eficácia (p<0,001) na prática, nas atitudes e na compreensão das pessoas, demonstrando o efeito positivo das orientações^(11)^.

Considera-se que o conhecimento sobre o processo de saúde-doença, a motivação, a autoeficácia, o suporte social e a compressão da condição clínica são elementos-chave para o aprimoramento das competências de autogestão^(12)^, especialmente após a cirurgia. O envolvimento da pessoa no cuidado, por meio do entendimento de sua enfermidade, da disposição para a mudança no estilo de vida e da rede de apoio, impacta diretamente a efetividade do cuidado em saúde^(13)^.

Assim, o uso de tecnologias educativas representa uma estratégia eficaz para ampliar o acesso a informações sobre a condição de saúde e os cuidados requeridos, favorecendo a compreensão e o protagonismo das pessoas no processo de recuperação. No contexto da CRVM, embora existam na literatura materiais educativos voltados aos cuidados pré e pós-operatórios, não foram identificadas cartilhas que abranjam todas as etapas do procedimento cirúrgico, contemplando orientações destinadas tanto aos pacientes quanto aos seus cuidadores. Logo, o fato justifica o desenvolvimento desta pesquisa, que partiu das seguintes questões: quais elementos devem integrar uma cartilha educativa com orientações voltadas ao cuidado de pessoas submetidas à cirurgia cardíaca? A cartilha elaborada apresenta evidências de validade de conteúdo e aparência?

### Relevância do estudo

Derivado da tese de doutorado intitulada O Enfermeiro e a Literacia em Saúde na autogestão do cuidado de pessoas pós-cirurgias cardíacas^(14)^, defendida na Universidade Federal do Paraná. Este estudo reforça a pertinência de tecnologias educativas baseadas em letramento em saúde, aplicadas ao cuidado de pessoas submetidas à revascularização miocárdica e de seus cuidadores.

## OBJETIVOS

Construir e validar uma cartilha com orientações de cuidados para pessoas submetidas à cirurgia cardíaca.

## MÉTODOS

Trata-se de pesquisa metodológica desenvolvida em quatro fases distintas: identificação das necessidades por meio de diagnóstico situacional; fundamentação teórica; elaboração e validação experimental do material educativo; e análise dos resultados^(15)^.

O estudo foi executado entre dezembro de 2020 e junho de 2021, na região Oeste do estado do Paraná, Brasil, em conformidade com as diretrizes do *Revised Standards for Quality Improvement Reporting Excellence* ^(16)^ vinculado à rede EQUATOR.

Esta investigação compõe a tese de doutorado intitulada “O enfermeiro e a literacia em saúde na autogestão do cuidado de pessoas pós-cirurgias cardíacas”^(14)^. A pesquisa foi aprovada por Comitê de Ética em Pesquisa (Parecer nº 5.048.079), e todos os participantes, incluindo os avaliadores especialistas, assinaram o Termo de Consentimento Livre e Esclarecido.

### Amostra, critérios de inclusão e exclusão

A coleta inicial dos dados foi realizada por meio de análise documental de prontuários clínicos arquivados no Serviço de Arquivo Médico e Estatístico (SAME) de um hospital referência em cardiologia do município de desenvolvimento do estudo. Foram incluídos os prontuários de pacientes diagnosticados com IAM e submetidos à CRVM entre julho e dezembro de 2019, conforme critérios pré-estabelecidos. Todos os casos que atenderam aos critérios definidos compuseram a amostra final, formada por 24 participantes.

Na etapa subsequente, foram incluídas as publicações brasileiras e internacionais em português, inglês e espanhol, sem limite de tempo e que contemplassem cuidados para todas as etapas da CRVM. Foram excluídos editoriais, livros, artigos de opinião, dissertações, teses e publicações duplicadas. Não foram identificados artigos que contemplassem o critério de inclusão.

A etapa seguinte foi a construção da cartilha, a qual ocorreu mediante os depoimentos das pessoas pós-CRVM dos últimos seis meses de 2019. Após a transcrição dos depoimentos, as principais dificuldades referidas foram agrupadas, servindo como base para a elaboração das orientações fundamentadas na literatura científica.

Na etapa final, foram selecionados juízes especialistas (mestres e doutores) e juízes técnicos (equipe multiprofissional do hospital de cardiologia), totalizando, no mínimo, seis juízes^(17)^. Os especialistas foram selecionados mediante amostragem por bola de neve, e os critérios de inclusão foram possuir experiência profissional mínima de quatro anos (clínica, ensino e/ou pesquisa), ser doutor ou mestre em enfermagem e residentes no país, sendo que tais critérios foram avaliados a partir dos dados no Currículo *Lattes*. Incluíram-se técnicos que atuavam no setor coronariano da instituição. Excluíram-se os juízes que não responderam ao formulário eletrônico no prazo indicado.

### Protocolo do estudo

O estudo foi desenvolvido em quatro etapas: (1) diagnóstico situacional; (2) procedimento teórico; (3) procedimento experimental; e (4) procedimento analítico^(14)^.

A primeira etapa identificou as necessidades e/ou dificuldades das pessoas submetidas à CRVM. A referida etapa foi realizada na primeira quinzena do mês de dezembro de 2020 em um hospital de referência em cardiologia no Oeste do Paraná, no SAME.

Após a aplicação dos critérios de elegibilidade, realizou-se a coleta de dados no domicílio com um instrumento que contemplava questões da percepção das pessoas da CRVM, desafios enfrentados pré e pós-cirurgia, instruções/orientações recebidas, preparação para alta e segmento dos cuidados da equipe de saúde no domicílio.

As entrevistas foram gravadas, transcritas e submetidas à análise por codificação temática, com apoio do *software Interface de R pour les Analyses Multidimensionnelles de Textes et de Questionnaires* 0.6-alpha 3, visando identificar as principais necessidades apontadas pelos participantes para subsidiar a construção da cartilha. Essa etapa seguiu as diretrizes do *COnsolidated criteria for REporting Qualitative research* ^(18)^.

A segunda etapa, revisão integrativa de literatura, ocorreu em julho de 2021, e para sua realização, foram seguidas as recomendações do *Preferred Reporting Systematic Reviews and Meta-Analyses of Studies* ^(19)^ nas bases de dados *Medical Literature Analysis and Retrieval System Online, Scientific Electronic Library Online*, Banco de Dados em Enfermagem e Literatura Latino-Americana e do Caribe em Ciências da Saúde. Após a seleção com base nos critérios de elegibilidade, as publicações foram avaliadas pela leitura do título e resumo, a fim de verificar se atendiam ao objetivo da revisão.

Na terceira etapa, foi elaborada a cartilha, concebida como tecnologia educativa de apoio, com o propósito de facilitar a aprendizagem e reforçar as informações verbais fornecidas pela equipe de saúde^(20)^. A construção textual do material foi orientada pelas necessidades e dificuldades relatadas pelos participantes nas entrevistas, as quais subsidiaram a definição dos conteúdos, com fundamento na literatura científica.

A quarta etapa consistiu na validação do conteúdo mediante técnica Delphi *online* modificada, tendo sido estabelecida a realização de uma reunião de consenso e no máximo três rodadas de avaliações. Nesta etapa, utilizou-se o formulário proposto por Rodrigues^(21)^, adaptado à temática da pesquisa, o qual contemplou aspectos sobre objetivo, estrutura, apresentação e relevância. Para os especialistas técnicos, o formulário contemplou questões sobre avaliação do conteúdo, linguagem, ilustração gráfica, motivação e adequação cultural. Em cada domínio avaliado, os juízes poderiam fazer considerações, caso julgassem necessário.

As respostas obedeciam ao formato de escala tipo Likert. Para os juízes especialistas, a escala Likert foi composta por cinco opções de respostas: 1 - discordo totalmente; 2 - concordo parcialmente; 3 - não concordo/nem discordo; 4 - concordo; e 5 - concordo totalmente. Para os juízes técnicos, a escala Likert possuía três escolhas: 0 - inadequado; 1 - parcialmente adequado; e 2 - adequado. Os instrumentos elaborados foram transpostos em formulários *online*, que viabilizou o acesso e avaliação simultâneas.

Os dados foram transcritos para um documento do programa *Microsoft Excel* ^®^ e, posteriormente, analisados por meio do Índice de Validade de Conteúdo (IVC), média, desvio padrão e alfa de Cronbach. Para a avaliação do IVC dos juízes especialistas, agruparam-se as respostas 1, 2 e 3 como “não concordo”, e as respostas 4 e 5 como “concordo”. As respostas dos juízes técnicos foram avaliadas considerando a somatória total dos pontos e a divisão desses pelo total de itens que compunham o questionário. Considerou-se o material como adequado quando 100% dos especialistas concordavam com o item^(22)^. Com vistas a atender às necessidades da instituição/rotina do hospital, optou-se utilizar duas escalas para a validação do instrumento com pontuações diferentes.

Os valores para o alfa de Cronbach ≥ 0,60 representam moderada consistência interna^(23)^. A cartilha foi considerada validada quando cada item obteve IVC^(23)^ igual ou superior a 0,8. Para a comparação entre a primeira e a segunda rodada, foi utilizado o teste de Wilcoxon. As fases de realização da pesquisa podem ser visualizadas na Figura 1 ^(16)^.

**Figura 1:** Fases da construção e validação da cartilha educativa, Curitiba, Paraná, Brasil, 2023

## RESULTADOS

Fizeram parte da primeira etapa 24 pessoas, sendo 18 (75%) homens que possuíam idade entre 44 e 68 anos, e 14 (58,3%) possuíam escolaridade menor do que nove anos de estudo. A transcrição dos áudios dos entrevistados teve 90,23% de aproveitamento, gerando 24 textos. A categorização do conteúdo revelou questionamentos relacionados aos hábitos alimentares no período pós-operatório, bem como lacunas informacionais na transição para o cuidado domiciliar, especialmente no que se refere às orientações para o autocuidado e na reabilitação.

A busca na literatura resultou em 43 artigos, sendo 40 inelegíveis. Os três artigos foram lidos na íntegra e posteriormente excluídos: dois por abordarem os cuidados de enfermagem no pós-operatório de cirurgia cardíaca na Unidade de Terapia Intensiva e ansiedade; e o terceiro por identificar as informações que os pacientes internados submetidos à CRVM acreditavam ser pertinentes receber antes da alta hospitalar em relação ao autocuidado^(24)^.

Desse modo, a revisão integrativa demostrou a necessidade de um material educativo que contemplasse todas as fases do processo cirúrgico: do pré-operatório até a reabilitação no domicílio. Logo, resultou na confecção do material educativo proposto, tendo como base fundamental as necessidades elencadas pelas pessoas que vivenciaram a experiência do processo cirúrgico e os desafios enfrentados por elas e seus cuidadores durante a reabilitação.

Para a construção da cartilha, utilizaram-se orientações propostas pelo Ministério da Saúde e pela Sociedade Brasileira de Cardiologia, que ocorreu por meio da elaboração textual, confecção das ilustrações e diagramação. Participaram da etapa um jornalista e um *design* gráfico. Confeccionou-se o material em tamanho de folha padrão A4, facilitando o manuseio e leitura. A fonte utilizada foi Lato, com tamanho 10, espessura regular, altura entre linhas 8 e parágrafos 16, e alinhamento justificado. A cor primária da cartilha foi a vermelha, com variação ao rosa-claro; a secundária foi verde, com variação ao verde-claro; a quaternária foi marrom, com variação ao marrom-claro; e as neutras foram preto, branco e cinza. Após a diagramação, a cartilha foi enviada para análise pelos especialistas, juízes e técnicos.

A editoração foi guiada pelo interesse do público-alvo. Buscou-se criar textos diretos utilizando uma linguagem clara e acessível. A versão da cartilha enviada pelos autores para a validação foi intitulada “Cirurgia Cardíaca - Cuidados Perioperatório Cardíacos: Cartilha de orientações para pessoas e cuidadores”.

A validação do conteúdo contou com a participação de sete especialistas, sendo esses majoritariamente do sexo feminino (85,7%), doutores (57,1%) e mestres em enfermagem (42,9%). O grupo de juízes técnicos foi composto por uma técnica de enfermagem que atuava no setor em que foi realizada a pesquisa e um fisioterapeuta especializado em reabilitação cardíaca intensiva. Os enfermeiros e médicos não responderam.

Os especialistas técnicos, após a análise do material, consideraram 100% adequado em todos os quesitos. Desta forma, não foi realizada segunda rodada com os participantes. Os resultados relacionados à concordância entre os juízes especialistas são apresentados na Tabela 1.

**Tabela 1:** Processo de validação de conteúdo do material, Curitiba, Paraná, Brasil, 2023

Itens avaliados	Questões	1ª rodada			2ª rodada		
		Média	Desvio padrão	IVC (%)	Média	Desvio padrão	IVC (%)
Conteúdo	1 - As informações/conteúdos são ou estão coerentes com as necessidades cotidianas dos cuidados com pessoas pós-cirurgias cardíacas?	3,9	1,3	71,4	4,7	0,5	100
	2 - As informações (e conteúdo) são importantes para a qualidade do cuidado das pessoas pós-cirurgia cardíaca.	4,0	1,7	71,4	4,6	0,5	100
	3 - As informações da tecnologia educativa convidam e/ou instigam a mudança de comportamento e atitude do paciente aos cuidados pós-cirurgias cardíacas.	4,3	1,1	85,7	4,4	0,5	100
	4 - Pode circular no meio científico da área.	4,7	0,5	100	4	1,0	86
	5 - Atende aos objetivos de instituições que atendem pessoas para a realização de cirurgia cardíaca.	4,4	0,5	100	4,7	0,4	100
Estrutura e apresentação	6 - A cartilha é apropriada para as pessoas que irão realizar cirurgia cardíaca.	4,1	1,1	85,7	4,6	0,5	100
	7 - As mensagens estão apresentadas de maneira clara e objetiva.	4,0	1,4	71,4	4,3	1,1	86
	8 - As informações apresentadas estão cientificamente corretas.	4,7	0,5	100	4,4	0,5	100
	9 - O material está apropriado ao nível sociocultural dos cuidados com pessoas pós-cirurgia cardíaca.	3,3	1,4	42,9	4,7	0,5	100
	10 - Há uma sequência lógica de conteúdo proposto.	4,7	0,5	100	4,7	0,5	100
	11 - As informações estão bem estruturadas em concordância e ortografia.	3,0	1,3	42,9	3,4	1,5	57
	12 - O estilo da redação corresponde ao nível de conhecimento dos pacientes e de suas famílias.	3,0	1,3	42,9	4,3	0,5	100
	13 - As informações da capa, contracapa, sumário, agradecimentos e/ou apresentação são coerentes.	3,1	1,5	42,9	4,4	0,5	100
	14 - O tamanho do título e dos tópicos está adequado.	4,4	0,5	100	4,1	1,1	100
	15 - As ilustrações estão expressivas e suficientes.	4,0	1,0	85,7	4	1	86
	16 - O número de páginas está adequado.	4,1	1,1	85,7	4,3	0,5	100
Relevância	17 - Os temas retratam aspectos-chave que devem ser reforçados.	4,4	0,5	100	4,6	0,5	100
	18 - O material permite a transferência e generalização do aprendizado a diferentes contextos (hospital e domicílio).	4,7	0,5	100	4,4	0,5	100
	19 - A cartilha propõe a construção de conhecimento.	4,7	0,5	100	4,6	0,5	100
	20 - O material aborda os assuntos necessários para o saber das pessoas pós-cirurgias cardíacas.	4,7	0,5	100	4,4	0,5	100
	21 - A cartilha está adequada para ser usada por qualquer pessoa que tenha sido submetida à cirurgia cardíaca.	4,4	0,5	100	4	1	86
IVC geral				82,3			94,6

*Legenda: teste de Wilcoxon (valor de p) = 0,052; IVC - Índice de Validade de Conteúdo.*

O alpha de Cronbach na segunda rodada foi de 0,93, representando um resultado relevante se comparado aos juízes especialistas. Em relação ao IVC, os resultados gerais foram de 83,3% e 94,6%, na primeira e segunda rodada, respectivamente. As principais sugestões dos juízes se encontram no Quadro 1, e foram relacionadas às questões 9, 11, 12 e 13.

**Quadro 1:** Recomendações dos especialistas de conteúdo fornecidas na primeira rodada de validação, Curitiba, Paraná, Brasil, 2023

Questão	Item analisado	Recomendação
9	O material está apropriado ao nível sociocultural dos cuidados com pessoas pós-cirurgia cardíaca.	Rever a terminologia técnica.
11	As informações estão bem estruturadas em concordância e ortografia.	Rever ortografia e concordância.
12	O estilo da redação corresponde ao nível de conhecimento das pessoas e de suas famílias.	Rever termos técnicos.
13	As informações da capa, contracapa, sumário, agradecimentos e/ou apresentação são coerentes.	Revisar as cores utilizadas, realizar mudança no título e no índice.

Após as adequações, foi realizada uma nova análise por sete juízes, e somente a questão 11 apresentou necessidade de revisão quanto à ortografia e concordância gramatical, sem interferências no resultado do IVC geral, que indicou boa validade de conteúdo com valor de 94,6%.

## DISCUSSÃO

A cartilha visa minimizar as lacunas de conhecimento e informar as pessoas submetidas à CRVM e na recuperação, além de constituir um instrumento de apoio para a equipe de saúde. Os aspectos considerados pelas pessoas submetidas à CRVM tardio foram similares a outros estudos^(24,25)^.

A construção da cartilha foi orientada por percepções dos próprios usuários, o que confere ao material uma abordagem personalizada e alinhada às dificuldades reais enfrentadas no pós-operatório da CRVM. Dessa forma, a partir dos relatos das pessoas submetidas à CRVM tardia e de seus cuidadores, elaborou-se material educativo que atendesse às necessidades dessa população fundamentada na literatura.

Salienta-se que, na reabilitação tardia, foram apontadas questões relativas à identificação dos fatores de risco, risco psicossocial e suporte de apoio. A comunicação ineficaz com os serviços de saúde emergiu como fator de distanciamento na relação entre os pacientes e equipe de saúde.

Durante a reabilitação tardia, destacaram-se questões relacionadas à labilidade emocional e limitações impostas pela CRVM, e o medo de um novo IAM, mesmo após um ano do evento. A presença de um plano de intervenção individualizado permite a definição de estratégias que viabilizam a prática do cuidado, sobretudo quando associado a ações educativas em saúde. Essa combinação favorece a adesão ao tratamento, amplia o conhecimento dos pacientes, aprimora o autocuidado e contribui para a compreensão do procedimento por meio de informações claras e fundamentadas^(26,27)^.

Segundo pesquisa americana, os materiais educativos voltados à saúde cardiovascular apresentam complexidade textual superior à capacidade de compreensão do público-alvo, implicando negativamente a interpretação das informações e dificultando sua aplicação na tomada de decisão relacionada ao autocuidado^(28)^.

Os obstáculos relacionados ao acesso a dados seguros e à interpretação das informações comunicadas criam obstáculos para alcançar a eficácia do LS. Portanto, é crucial criar materiais didáticos em linguagem compreensível e sua validação pelo público-alvo, a fim de minimizar tais obstáculos e tornar o conteúdo compreensível^(28)^.

O nível de LS influencia a compreensão das informações, a aptidão para executar ações relacionadas ao cuidado, o comprometimento com o plano terapêutico e a aceitação de ações de promoção à saúde^(29,30)^. Representa um fator-chave para a mudança do estilo de vida e promoção de resultados mais favoráveis à saúde, além de ser um fator determinante para modificação de hábitos e, como consequência, para aquisição de melhores resultados relacionados à saúde^(31,32)^.

Portanto, a adequação e a clareza das informações, aliadas às habilidades da equipe de saúde na comunicação, são essenciais para pessoas com baixos níveis LS, e a utilização de tecnologias na área da saúde pode auxiliar no processo^(33)^. Nesse sentido, revisão de escopo buscou classificar e mapear as características e intervenções que tornam a equipe de saúde responsiva à LS do paciente, identificando 19 estratégias, destacando-se o uso de materiais educativos^(34)^. Sendo assim, a confecção e divulgação de um material educativo direcionado a pessoas pós-CRVM e seus cuidadores são um recurso didático que contribui para o aumento do nível de LS, ao apresentar orientações de forma objetiva, com linguagem acessível e por meio de imagens.

A técnica Delphi empregada para validação do conteúdo permitiu a participação especialistas de maneira remota^(35)^, resultando no desenvolvimento efetivo, prático e confiável de um material educativo.

A cartilha pode estimular o aprendizado, contribuir para a recuperação pós-cirurgia e apoiar o enfrentamento dos desafios relacionados à reabilitação. Houve consenso quanto à adequação do material ao processo de ensino em saúde, por apresentar conteúdo acessível, pertinente e formato apropriado^(36)^.

O processo de avaliação por especialistas confere rigor metodológico à construção do conteúdo educativo, contribuindo para seu aprimoramento e assegurando maior efetividade em relação ao proposito a que se destina^(37,38)^.

### Limitações do estudo

O fator considerado limitante nesta pesquisa consistiu na obtenção dos dados restritos a um serviço de saúde local, remetendo as necessidades daquelas pessoas submetidas à CRVM.

### Contribuições para as áreas da enfermagem, saúde ou políticas públicas

O material resultante desta pesquisa aborda os principais tópicos que devem ser enfatizados durante as orientações para pessoas que serão e/ou foram submetidas à revascularização miocárdica e seus cuidadores, as quais foram elencadas a partir das necessidades dos pacientes e validadas por profissionais especialistas na temática.

A cartilha pode ser incorporada na prática clínica do enfermeiro durante a consulta de enfermagem, atuando como instrumento de apoio para orientação rápida e qualificada e contribuindo para a redução de intercorrência no pós-operatório de cirurgia cardíaca. A construção e validação de materiais educativos nos serviços de saúde são essenciais, por proporcionarem informações seguras que auxiliam as pessoas a sanarem suas dúvidas.

A escuta qualificada da pessoa submetida à CRVM, aliada à fundamentação teórica baseada na literatura, resultou na elaboração de uma cartilha cujo conteúdo foi validado por especialistas. A tecnologia educativa desenvolvida apresentou evidências de validade de conteúdo, sendo recomendada para uso por profissionais de saúde, com ênfase na atuação de enfermagem. Sua utilização também se mostra adequada no contexto do cuidado prestado entre familiares e pacientes, pois contempla orientações desde o período pré-operatório até a fase de reabilitação domiciliar.

Verificou-se que a validação pelos especialistas e da equipe de saúde em sua construção qualificou a cartilha para uso na instituição. A elaboração do material evidencia resultados promissores relacionados à assistência oferecida às pessoas submetidas à CRVM. Conforme o rigor do processo de validação e as adequações necessárias, a cartilha apresenta potencial para ser implementada em diferentes contextos assistenciais que atendam indivíduos com esse perfil clínico.

## Data Availability

Os dados de pesquisa estão disponíveis em repositório: https://doi.org/10.5281/zenodo.17214413.
